# Dimethyl 7-meth­oxy­tetra­cyclo­[6.4.0.0^2,4^.0^3,7^]dodeca-1(12),5,8,10-tetra­ene-3,4-dicarboxyl­ate

**DOI:** 10.1107/S1600536812037233

**Published:** 2012-09-05

**Authors:** Amin Moazeni, Christopher O. Bender, René T. Boeré

**Affiliations:** a Department of Chemistry and Biochemistry, University of Lethbridge, Lethbridge, AB, Canada T1K 3M4

## Abstract

The title compound, C_17_H_16_O_5_, is a previously unreported substituted semibulvalene cage compound (that is, a tricyclic hydro­carbon formed from one cyclo­propane and two cyclo­pentene rings which also has one double bond fused to a benzene ring). It has one meth­oxy substituent attached to the bridgehead C atom that links only the two cyclo­pentene rings and two methyl carboxyl­ate groups located on the C atom shared by all three non-benzene rings and that shared only between the cyclo­propane and the cyclo­pentene rings. The stereochemistry of the two enanti­omers (racemate) that assemble in each unit cell is *RRRS* and *SSSR*. In the crystal, mol­ecules are linked *via* C—H⋯O hydrogen bonds and C—H⋯π inter­actions, forming double-layered sheets lying perpendicular to the *a* axis.

## Related literature

For general background, see: Bender & Brooks (1975 ▶). For related structures, see: Muneer *et al.* (1997 ▶); Pokkuluri, Scheffer & Trotter (1994 ▶); Pokkuluri, Scheffer, Trotter & Yap (1994 ▶). For a description of the Cambridge Structural Database, see: Allen (2002 ▶).
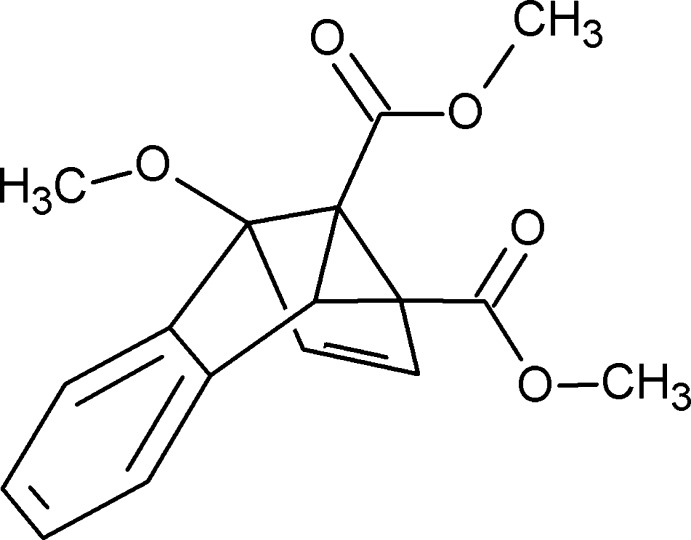


## Experimental

### 

#### Crystal data

C_17_H_16_O_5_
*M* *_r_* = 300.30
Monoclinic, 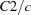

*a* = 23.892 (2) Å
*b* = 7.9999 (8) Å
*c* = 15.4182 (15) Å
β = 90.028 (1)°
*V* = 2946.9 (5) Å^3^
*Z* = 8
Mo *K*α radiation
μ = 0.10 mm^−1^
*T* = 173 K
0.37 × 0.33 × 0.28 mm

#### Data collection

Bruker APEXII CCD diffractometer
Absorption correction: multi-scan (*SADABS*; Bruker, 2008 ▶) *T*_min_ = 0.672, *T*_max_ = 0.746
19005 measured reflections
3005 independent reflections
2607 reflections with *I* > 2σ(*I*)
*R*_int_ = 0.029

#### Refinement

*R*[*F* ^2^ > 2σ(*F* ^2^)] = 0.034
*wR*(*F* ^2^) = 0.094
*S* = 1.07
3005 reflections
202 parameters
H-atom parameters constrained
Δρ_max_ = 0.31 e Å^−3^
Δρ_min_ = −0.21 e Å^−3^

### 

Data collection: *APEX2* (Bruker, 2008 ▶); cell refinement: *SAINT-Plus* (Bruker, 2008 ▶); data reduction: *SAINT-Plus*; program(s) used to solve structure: *SHELXS97* (Sheldrick, 2008 ▶); program(s) used to refine structure: *SHELXTL* (Sheldrick, 2008 ▶); molecular graphics: *Mercury* (Macrae *et al.*, 2008 ▶); software used to prepare material for publication: *publCIF* (Westrip, 2010 ▶).

## Supplementary Material

Crystal structure: contains datablock(s) I, global. DOI: 10.1107/S1600536812037233/hg5246sup1.cif

Structure factors: contains datablock(s) I. DOI: 10.1107/S1600536812037233/hg5246Isup2.hkl

Supplementary material file. DOI: 10.1107/S1600536812037233/hg5246Isup3.cml

Additional supplementary materials:  crystallographic information; 3D view; checkCIF report

## Figures and Tables

**Table 1:** Hydrogen-bond geometry (Å, °)

*D*—H⋯*A*	*D*—H	H⋯*A*	*D*⋯*A*	*D*—H⋯*A*
C6—H6⋯O2^i^	0.95	2.53	3.3895 (16)	151
C10—H10⋯O5^ii^	0.95	2.54	3.2425 (15)	131
C7—H7⋯C9^iii^	0.95	2.86	3.773 (2)	162

Symmetry codes: (i) 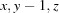; (ii) 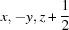; (iii) 
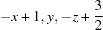
.

**Fig. 1.**
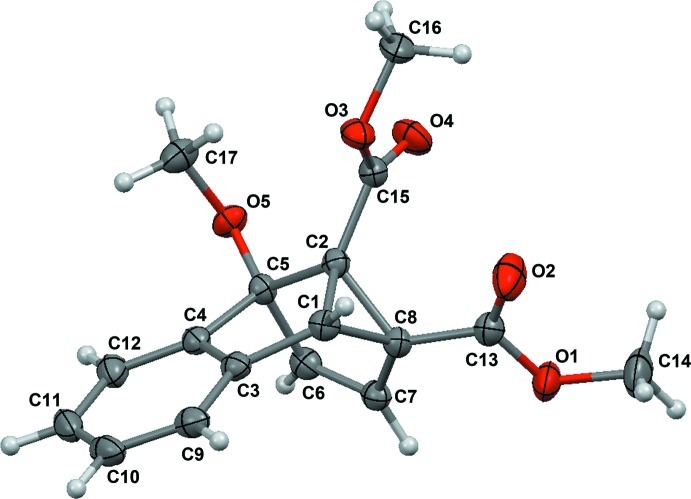
The molecular structure of (I) with displacement ellipsoids drawn at the 50% probability level. H atoms are shown as spheres of arbitrary radius.

**Fig. 2.**
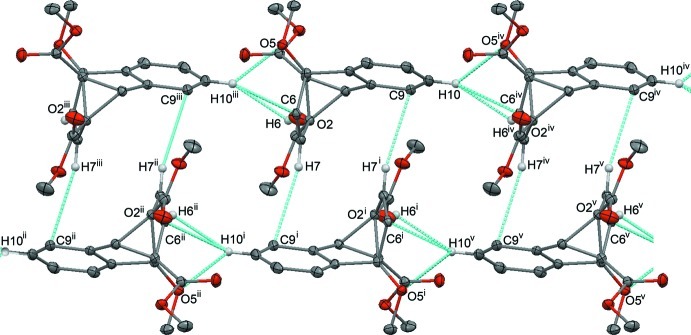
Crystal plot viewed down the b axis (c axis horizontal) displaying H···O, H···C and H···H contacts less than the sum of the v. d. Waals' radii [Symmetry codes: (i) 1 - x, y, 1.5 - z; (ii) 1 - x, -y, 1 - z; (iii) x, -y, -0.5 + z; (iv) x, -y, 0.5 - z; (v) 1 - x, -y, 2 - z.] Displacement ellipsoids are drawn at the 30% probability level; only those H atoms involved in short contacts are included and are drawn as spheres of arbitrary radius. Short contacts are drawn as dashed tubes.

**Fig. 3.**
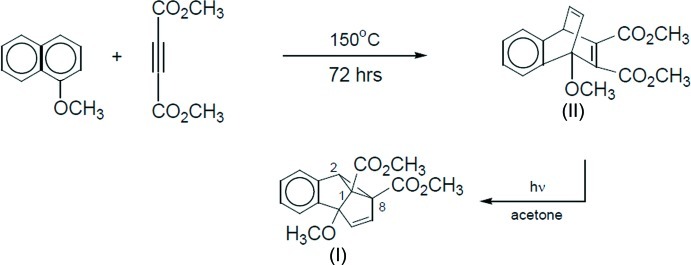
Scheme showing the photochemical preparation of (I) from the barrelene (II) as well as other preparative chemical steps.

## supplementary crystallographic information

### Comment

The structure of the title compound (I) is shown in Fig. 1. Although a number of bulvalene (two fused benzene rings) and semibulvalene (one fused benzene) structures have previously been reported, three have features that make comparison to (I) particularly meaningful. The bulvalene 8*c*,8*d*-dibenzoyl-4*b*-methoxy-4*b*,8*b*,8*c*,8*d*-tetrahydrodibenzo(*a,f*)cyclopropa(*cd*)pentalene, CSD (Allen, 2002) refcode: ROHFAV, has a methoxy group in the same bridgehead location as in the title compound (Muneer *et al.*, 1997). Furthermore, the two sites on the cyclopropane ring are also substituted, albeit with phenyl ketone groups in place of the esters. The semibulvalene methyl 6*c*-benzoyl-2*a*,2*b*,6*b*,6*c*-tetrahydrobenzo(*a*)cyclopropa(*cd*)pentalene-6*b*-carboxylate has a methyl ester functional group in place of the methoxy at the bridgehead C in (I) and has one phenyl ketone group attached at the cyclopropane ring (CSD refcode: LEKLES; Pokkuluri, Scheffer, Trotter & Yap, 1994). The semibulvalene dimethyl 3,8-diphenyl-2*a*,2*b*,8*b*,8*c*-tetrahydrocyclopropa(1',2',3':3,3*a*,4) pentaleno(1,2 - *b*)naphthalene-2*a*,8*c*-dicarboxylate shares with (I) the substitution of two methyl ester groups in the same locations (CSD refcode: LEKLIW; Pokkuluri, Scheffer, & Trotter, 1994). This structure differs by not having a bridgehead methoxy group and that the fused aromatic ring is a diphenyl-substituted naphthalene ring in place of the unsubstituted benzene ring in (I). A comparison of the metric parameters common to these four structures shows great similarity with only a few values deviating by more than 1%.

The five short intermolecular contacts in the crystal lattice of (I) are displayed in Fig. 2 looking down the *b* axis with *c* horizontal. The contacts extend in such a way as to develop a "double layer" parallel to the *bc* planes. The contact distances are O2···H6', 2.530 (1); O5···H10' 2.5353 (8); C6···H10', 2.810 (1); H6···H10', 2.3578 (2) and H7···C9', 2.9307 (2) Å. The interactions involving O2 and O5 are sufficient to develop the sheet structure; the doubling of the sheets exclusively involves "T-shaped" interactions between H7 and C9.

### Experimental

The title compound (I) is the major product from the acetone sensitized irradiation of dimethyl 1-methoxy-4-hydro-1,4-ethenonaphthalene-2,3-dicarboxylate (II). The melting range of (I) is 382–384 K. The barrelene (II) was of interest in connection with a study of polar substituents in pericyclic reactions (Bender *et al.*, 1975) and was synthesized from the Diels-Alder reaction between 1-methoxynaphthalene and dimethyl acetylenedicarboxylate, as outlined in Figure 3.

### Refinement

All hydrogen atoms were located on a difference map. Hydrogen atoms attached to carbon are treated as riding, with C—H = 0.98 Å and *U*_iso_(H) = 1.5*U*_eq_(C) for methyl, C—H = 1.00 Å and *U*_iso_(H) = 1.2*U*_eq_(C) for methine and C—H = 0.95 Å and *U*_iso_(H) = 1.2*U*_eq_(C) for aromatic and alkene H atoms. The highest residual peak is only a fraction of the electron density of a single H atom, 0.31 e.Å^-3^, and is located between C1 and C5.

### Crystal data

**Table:**

C_17_H_16_O_5_	*F*(000) = 1264
*M**_r_* = 300.30	*D*_x_ = 1.354 Mg m^−^^3^
Monoclinic, *C*2/*c*	Mo *K*α radiation, λ = 0.71073 Å
Hall symbol: -C 2yc	Cell parameters from 9907 reflections
*a* = 23.892 (2) Å	θ = 2.6–28.8°
*b* = 7.9999 (8) Å	µ = 0.10 mm^−^^1^
*c* = 15.4182 (15) Å	*T* = 173 K
β = 90.028 (1)°	Block, colourless
*V* = 2946.9 (5) Å^3^	0.37 × 0.33 × 0.28 mm
*Z* = 8	

### Data collection

**Table:**

Bruker APEXII CCD diffractometer	3005 independent reflections
Radiation source: fine-focus sealed tube, Bruker D8	2607 reflections with *I* > 2σ(*I*)
Graphite monochromator	*R*_int_ = 0.029
Detector resolution: 66.06 pixels mm^-1^	θ_max_ = 26.4°, θ_min_ = 1.7°
φ and ω scans	*h* = −29→29
Absorption correction: multi-scan (*SADABS*; Bruker, 2008)	*k* = −9→9
*T*_min_ = 0.672, *T*_max_ = 0.746	*l* = −19→19
19005 measured reflections	

### Refinement

**Table:**

Refinement on *F*^2^	Primary atom site location: structure-invariant direct methods
Least-squares matrix: full	Secondary atom site location: difference Fourier map
*R*[*F*^2^ > 2σ(*F*^2^)] = 0.034	Hydrogen site location: inferred from neighbouring sites
*wR*(*F*^2^) = 0.094	H-atom parameters constrained
*S* = 1.07	*w* = 1/[σ^2^(*F*_o_^2^) + (0.0508*P*)^2^ + 1.467*P*] where *P* = (*F*_o_^2^ + 2*F*_c_^2^)/3
3005 reflections	(Δ/σ)_max_ = 0.001
202 parameters	Δρ_max_ = 0.31 e Å^−^^3^
0 restraints	Δρ_min_ = −0.21 e Å^−^^3^

### Special details

**Table:**

Experimental. A crystal coated in Paratone (TM) oil was mounted on the end of a thin glass capillary and cooled in the gas stream of the diffractometer Kryoflex device.
Geometry. All e.s.d.'s (except the e.s.d. in the dihedral angle between two l.s. planes) are estimated using the full covariance matrix. The cell e.s.d.'s are taken into account individually in the estimation of e.s.d.'s in distances, angles and torsion angles; correlations between e.s.d.'s in cell parameters are only used when they are defined by crystal symmetry. An approximate (isotropic) treatment of cell e.s.d.'s is used for estimating e.s.d.'s involving l.s. planes.
Refinement. Refinement of *F*^2^ against ALL reflections. The weighted *R*-factor *wR* and goodness of fit *S* are based on *F*^2^, conventional *R*-factors *R* are based on *F*, with *F* set to zero for negative *F*^2^. The threshold expression of *F*^2^ > σ(*F*^2^) is used only for calculating *R*-factors(gt) *etc*. and is not relevant to the choice of reflections for refinement. *R*-factors based on *F*^2^ are statistically about twice as large as those based on *F*, and *R*- factors based on ALL data will be even larger.

### Fractional atomic coordinates and isotropic or equivalent isotropic displacement parameters (Å^2^)

**Table:**

	*x*	*y*	*z*	*U*_iso_*/*U*_eq_	
C3	0.38022 (5)	0.25083 (15)	0.81435 (7)	0.0222 (3)	
C6	0.42265 (5)	0.09543 (15)	0.64775 (7)	0.0226 (3)	
H6	0.4317	−0.0146	0.6294	0.027*	
C7	0.45963 (5)	0.21768 (15)	0.65739 (7)	0.0225 (2)	
H7	0.4990	0.2036	0.6525	0.027*	
C2	0.38959 (5)	0.39617 (14)	0.75702 (7)	0.0216 (2)	
H2	0.3809	0.5104	0.7796	0.026*	
C17	0.26658 (5)	0.11991 (17)	0.64184 (9)	0.0315 (3)	
H17A	0.2585	0.0638	0.6970	0.047*	
H17B	0.2406	0.0796	0.5973	0.047*	
H17C	0.2620	0.2409	0.6489	0.047*	
C4	0.36095 (5)	0.11440 (14)	0.76658 (7)	0.0217 (2)	
C12	0.34945 (5)	−0.03788 (16)	0.80631 (8)	0.0285 (3)	
H12	0.3377	−0.1319	0.7733	0.034*	
C16	0.27099 (6)	0.67723 (17)	0.60114 (10)	0.0359 (3)	
H16A	0.2966	0.7588	0.5752	0.054*	
H16B	0.2436	0.7356	0.6374	0.054*	
H16C	0.2515	0.6162	0.5551	0.054*	
C8	0.43190 (5)	0.37858 (15)	0.67664 (7)	0.0219 (3)	
C15	0.33539 (5)	0.45632 (14)	0.60978 (7)	0.0226 (3)	
C13	0.45429 (5)	0.54272 (15)	0.64747 (8)	0.0264 (3)	
C11	0.35563 (5)	−0.04820 (18)	0.89585 (9)	0.0339 (3)	
H11	0.3468	−0.1496	0.9248	0.041*	
C1	0.36939 (5)	0.34981 (14)	0.66910 (7)	0.0198 (2)	
C9	0.38762 (5)	0.23822 (17)	0.90355 (8)	0.0283 (3)	
H9	0.4012	0.3302	0.9363	0.034*	
C5	0.36397 (5)	0.15424 (14)	0.66990 (7)	0.0204 (2)	
C10	0.37463 (5)	0.08795 (19)	0.94345 (8)	0.0339 (3)	
H10	0.3788	0.0778	1.0045	0.041*	
C14	0.53073 (7)	0.6835 (2)	0.58505 (12)	0.0471 (4)	
H14A	0.5057	0.7368	0.5429	0.071*	
H14B	0.5668	0.6595	0.5575	0.071*	
H14C	0.5365	0.7587	0.6344	0.071*	
O5	0.32282 (3)	0.08404 (11)	0.61609 (5)	0.0250 (2)	
O4	0.33743 (4)	0.44688 (12)	0.53199 (6)	0.0350 (2)	
O3	0.30236 (4)	0.56102 (11)	0.65372 (6)	0.0275 (2)	
O1	0.50578 (4)	0.52885 (12)	0.61514 (6)	0.0344 (2)	
O2	0.42898 (4)	0.67223 (12)	0.65173 (8)	0.0458 (3)	

### Atomic displacement parameters (Å^2^)

**Table:**

	*U*^11^	*U*^22^	*U*^33^	*U*^12^	*U*^13^	*U*^23^
C3	0.0184 (5)	0.0274 (6)	0.0207 (6)	0.0024 (5)	0.0014 (4)	0.0011 (4)
C6	0.0257 (6)	0.0226 (6)	0.0195 (5)	0.0039 (5)	0.0025 (4)	−0.0009 (4)
C7	0.0216 (6)	0.0256 (6)	0.0204 (5)	0.0029 (5)	0.0020 (4)	0.0028 (4)
C2	0.0227 (6)	0.0220 (6)	0.0201 (6)	0.0017 (4)	0.0005 (4)	−0.0021 (4)
C17	0.0227 (6)	0.0329 (7)	0.0389 (7)	−0.0019 (5)	−0.0017 (5)	−0.0055 (6)
C4	0.0191 (5)	0.0239 (6)	0.0221 (6)	0.0024 (4)	0.0020 (4)	0.0025 (4)
C12	0.0254 (6)	0.0253 (6)	0.0346 (7)	0.0020 (5)	0.0043 (5)	0.0061 (5)
C16	0.0276 (7)	0.0304 (7)	0.0496 (8)	0.0063 (5)	−0.0019 (6)	0.0148 (6)
C8	0.0209 (6)	0.0232 (6)	0.0215 (6)	−0.0009 (5)	0.0006 (4)	0.0014 (4)
C15	0.0219 (6)	0.0222 (6)	0.0237 (6)	−0.0012 (5)	0.0007 (4)	0.0036 (4)
C13	0.0255 (6)	0.0247 (6)	0.0289 (6)	−0.0027 (5)	−0.0010 (5)	0.0025 (5)
C11	0.0270 (6)	0.0380 (7)	0.0366 (7)	0.0060 (6)	0.0071 (5)	0.0190 (6)
C1	0.0205 (5)	0.0196 (5)	0.0194 (5)	0.0001 (4)	0.0011 (4)	0.0000 (4)
C9	0.0223 (6)	0.0419 (7)	0.0205 (6)	0.0028 (5)	0.0005 (4)	0.0001 (5)
C5	0.0219 (6)	0.0191 (5)	0.0201 (6)	−0.0001 (4)	0.0001 (4)	−0.0005 (4)
C10	0.0252 (6)	0.0540 (9)	0.0226 (6)	0.0073 (6)	0.0027 (5)	0.0121 (6)
C14	0.0404 (8)	0.0356 (8)	0.0654 (10)	−0.0129 (7)	0.0139 (7)	0.0118 (7)
O5	0.0227 (4)	0.0273 (4)	0.0251 (4)	−0.0022 (3)	−0.0014 (3)	−0.0064 (3)
O4	0.0426 (6)	0.0401 (6)	0.0223 (5)	0.0058 (4)	−0.0014 (4)	0.0062 (4)
O3	0.0257 (4)	0.0261 (4)	0.0309 (5)	0.0075 (3)	0.0013 (4)	0.0053 (3)
O1	0.0273 (5)	0.0291 (5)	0.0468 (6)	−0.0052 (4)	0.0079 (4)	0.0083 (4)
O2	0.0387 (6)	0.0227 (5)	0.0760 (8)	0.0002 (4)	0.0146 (5)	0.0079 (5)

### Geometric parameters (Å, º)

**Table:**

C3—C9	1.3902 (17)	C16—H16B	0.9800
C3—C4	1.3948 (17)	C16—H16C	0.9800
C3—C2	1.4776 (16)	C8—C13	1.4874 (16)
C6—C7	1.3262 (17)	C8—C1	1.5157 (16)
C6—C5	1.5179 (15)	C15—O4	1.2028 (15)
C6—H6	0.9500	C15—O3	1.3356 (14)
C7—C8	1.4778 (16)	C15—C1	1.4906 (16)
C7—H7	0.9500	C13—O2	1.2014 (16)
C2—C1	1.4857 (16)	C13—O1	1.3323 (15)
C2—C8	1.6060 (16)	C11—C10	1.389 (2)
C2—H2	1.0000	C11—H11	0.9500
C17—O5	1.4304 (15)	C1—C5	1.5699 (16)
C17—H17A	0.9800	C9—C10	1.3856 (19)
C17—H17B	0.9800	C9—H9	0.9500
C17—H17C	0.9800	C5—O5	1.4035 (14)
C4—C12	1.3910 (17)	C10—H10	0.9500
C4—C5	1.5260 (15)	C14—O1	1.4495 (16)
C12—C11	1.3908 (19)	C14—H14A	0.9800
C12—H12	0.9500	C14—H14B	0.9800
C16—O3	1.4432 (15)	C14—H14C	0.9800
C16—H16A	0.9800		
			
C9—C3—C4	120.49 (11)	C1—C8—C2	56.75 (7)
C9—C3—C2	129.03 (11)	O4—C15—O3	124.71 (11)
C4—C3—C2	110.48 (10)	O4—C15—C1	123.62 (11)
C7—C6—C5	111.19 (10)	O3—C15—C1	111.66 (9)
C7—C6—H6	124.4	O2—C13—O1	123.86 (11)
C5—C6—H6	124.4	O2—C13—C8	124.31 (11)
C6—C7—C8	111.48 (10)	O1—C13—C8	111.83 (10)
C6—C7—H7	124.3	C10—C11—C12	120.81 (12)
C8—C7—H7	124.3	C10—C11—H11	119.6
C3—C2—C1	107.47 (10)	C12—C11—H11	119.6
C3—C2—C8	119.23 (9)	C2—C1—C15	126.43 (10)
C1—C2—C8	58.56 (7)	C2—C1—C8	64.69 (8)
C3—C2—H2	118.6	C15—C1—C8	119.80 (9)
C1—C2—H2	118.6	C2—C1—C5	105.57 (9)
C8—C2—H2	118.6	C15—C1—C5	121.98 (10)
O5—C17—H17A	109.5	C8—C1—C5	103.41 (9)
O5—C17—H17B	109.5	C10—C9—C3	118.28 (12)
H17A—C17—H17B	109.5	C10—C9—H9	120.9
O5—C17—H17C	109.5	C3—C9—H9	120.9
H17A—C17—H17C	109.5	O5—C5—C6	112.94 (9)
H17B—C17—H17C	109.5	O5—C5—C4	117.42 (9)
C12—C4—C3	121.20 (11)	C6—C5—C4	101.49 (9)
C12—C4—C5	128.50 (11)	O5—C5—C1	116.86 (9)
C3—C4—C5	109.67 (10)	C6—C5—C1	103.35 (9)
C11—C12—C4	117.92 (12)	C4—C5—C1	102.69 (9)
C11—C12—H12	121.0	C9—C10—C11	121.25 (12)
C4—C12—H12	121.0	C9—C10—H10	119.4
O3—C16—H16A	109.5	C11—C10—H10	119.4
O3—C16—H16B	109.5	O1—C14—H14A	109.5
H16A—C16—H16B	109.5	O1—C14—H14B	109.5
O3—C16—H16C	109.5	H14A—C14—H14B	109.5
H16A—C16—H16C	109.5	O1—C14—H14C	109.5
H16B—C16—H16C	109.5	H14A—C14—H14C	109.5
C7—C8—C13	123.16 (10)	H14B—C14—H14C	109.5
C7—C8—C1	107.10 (9)	C5—O5—C17	114.42 (9)
C13—C8—C1	117.74 (10)	C15—O3—C16	115.20 (10)
C7—C8—C2	120.91 (9)	C13—O1—C14	115.38 (11)
C13—C8—C2	112.49 (10)		
			
C5—C6—C7—C8	−6.71 (14)	C7—C8—C1—C2	116.16 (10)
C9—C3—C2—C1	−173.72 (11)	C13—C8—C1—C2	−99.91 (11)
C4—C3—C2—C1	5.71 (13)	C7—C8—C1—C15	−124.75 (11)
C9—C3—C2—C8	123.07 (13)	C13—C8—C1—C15	19.19 (15)
C4—C3—C2—C8	−57.51 (14)	C2—C8—C1—C15	119.09 (12)
C9—C3—C4—C12	−0.96 (18)	C7—C8—C1—C5	15.07 (11)
C2—C3—C4—C12	179.56 (10)	C13—C8—C1—C5	159.00 (10)
C9—C3—C4—C5	−172.58 (10)	C2—C8—C1—C5	−101.09 (9)
C2—C3—C4—C5	7.94 (13)	C4—C3—C9—C10	−0.89 (17)
C3—C4—C12—C11	2.51 (18)	C2—C3—C9—C10	178.49 (11)
C5—C4—C12—C11	172.41 (11)	C7—C6—C5—O5	143.12 (10)
C6—C7—C8—C13	−147.41 (11)	C7—C6—C5—C4	−90.27 (11)
C6—C7—C8—C1	−5.91 (13)	C7—C6—C5—C1	15.91 (12)
C6—C7—C8—C2	55.12 (14)	C12—C4—C5—O5	42.13 (16)
C3—C2—C8—C7	2.50 (16)	C3—C4—C5—O5	−147.04 (10)
C1—C2—C8—C7	−91.00 (12)	C12—C4—C5—C6	−81.48 (14)
C3—C2—C8—C13	−157.18 (11)	C3—C4—C5—C6	89.36 (11)
C1—C2—C8—C13	109.32 (11)	C12—C4—C5—C1	171.82 (11)
C3—C2—C8—C1	93.50 (11)	C3—C4—C5—C1	−17.34 (12)
C7—C8—C13—O2	169.30 (13)	C2—C1—C5—O5	150.13 (9)
C1—C8—C13—O2	31.53 (18)	C15—C1—C5—O5	−4.12 (15)
C2—C8—C13—O2	−31.55 (17)	C8—C1—C5—O5	−142.81 (9)
C7—C8—C13—O1	−10.10 (16)	C2—C1—C5—C6	−85.17 (10)
C1—C8—C13—O1	−147.87 (11)	C15—C1—C5—C6	120.58 (11)
C2—C8—C13—O1	149.05 (10)	C8—C1—C5—C6	−18.11 (11)
C4—C12—C11—C10	−2.25 (19)	C2—C1—C5—C4	20.10 (11)
C3—C2—C1—C15	136.43 (11)	C15—C1—C5—C4	−134.15 (10)
C8—C2—C1—C15	−109.52 (12)	C8—C1—C5—C4	87.16 (10)
C3—C2—C1—C8	−114.05 (10)	C3—C9—C10—C11	1.14 (18)
C3—C2—C1—C5	−16.31 (12)	C12—C11—C10—C9	0.5 (2)
C8—C2—C1—C5	97.74 (9)	C6—C5—O5—C17	172.52 (10)
O4—C15—C1—C2	150.54 (12)	C4—C5—O5—C17	54.92 (14)
O3—C15—C1—C2	−30.10 (15)	C1—C5—O5—C17	−67.78 (13)
O4—C15—C1—C8	71.47 (16)	O4—C15—O3—C16	−4.97 (17)
O3—C15—C1—C8	−109.17 (11)	C1—C15—O3—C16	175.68 (10)
O4—C15—C1—C5	−60.80 (16)	O2—C13—O1—C14	0.5 (2)
O3—C15—C1—C5	118.56 (11)	C8—C13—O1—C14	179.94 (12)

### Hydrogen-bond geometry (Å, º)

**Table:**

*D*—H···*A*	*D*—H	H···*A*	*D*···*A*	*D*—H···*A*
C6—H6···O2^i^	0.95	2.53	3.3895 (16)	151
C10—H10···O5^ii^	0.95	2.54	3.2425 (15)	131
C7—H7···C9^iii^	0.95	2.86	3.773 (2)	162

Symmetry codes: (i) *x*, *y*−1, *z*; (ii) *x*, −*y*, *z*+1/2; (iii) −*x*+1, *y*, −*z*+3/2.
